# Additional non-sentinel lymph node metastases in early oral cancer patients with positive sentinel lymph nodes

**DOI:** 10.1007/s00405-016-4280-2

**Published:** 2016-08-25

**Authors:** Inne J. Den Toom, Elisabeth Bloemena, Stijn van Weert, K. Hakki Karagozoglu, Otto S. Hoekstra, Remco de Bree

**Affiliations:** 1 Department of Otolaryngology-Head and Neck Surgery, VU University Medical Center, P.O. Box 7057, 1007 MB Amsterdam, The Netherlands; 2 Department of Oral and Maxillofacial Surgery/Oral Pathology, VU University Medical Center/Academic Center for Dentistry (ACTA) Amsterdam, P.O. Box 7057, 1007 MB Amsterdam, The Netherlands; 3 Department of Radiology and Nuclear Medicine, VU University Medical Center, P.O. Box 7057, 1007 MB Amsterdam, The Netherlands; 4 Department of Pathology, VU University Medical Center, P.O. Box 7057, 1007 MB Amsterdam, The Netherlands; 5 Department of Head and Neck Surgical Oncology, UMC Utrecht Cancer Center, University Medical Center Utrecht, P.O. Box 85500, 3508 GA Utrecht, The Netherlands

**Keywords:** Sentinel lymph node biopsy, Mouth neoplasms, Neck dissection, Lymph nodes, Lymphatic metastasis, Neoplasm micrometastasis

## Abstract

To determine risk factors for additional non-sentinel lymph node metastases in neck dissection specimens of patients with early stage oral cancer and a positive sentinel lymph node biopsy (SLNB). A retrospective analysis of 36 previously untreated SLNB positive patients in our institution and investigation of currently available literature of positive SLNB patients in early stage oral cancer was done. Degree of metastatic involvement [classified as isolated tumor cells (ITC), micro- and macrometastasis] of the sentinel lymph node (SLN), the status of other SLNs, and additional non-SLN metastases in neck dissection specimens were analyzed. Of 27 studies, comprising 511 patients with positive SLNs, the pooled prevalence of non-SLN metastasis in patients with positive SLNs was 31 %. Non-SLN metastases were detected (available from 9 studies) in 13, 20, and 40 % of patients with ITC, micro-, and macrometastasis in the SLN, respectively. The probability of non-SLN metastasis seems to be higher in the case of more than one positive SLN (29 vs. 24 %), the absence of negative SLNs (40 vs. 19 %), and a positive SLN ratio of more than 50 % (38 vs. 19 %). Additional non-SLN metastases were found in 31 % of neck dissections following positive SLNB. The presence of multiple positive SLNs, the absence of negative SLNs, and a positive SLN ratio of more than 50 % may be predictive factors for non-SLN metastases. Classification of SLNs into ITC, micro-, and macrometastasis in the future SLNB studies is important to answer the question if treatment of the neck is always needed after positive SLNB.

## Introduction

Sentinel lymph node biopsy (SLNB) has been introduced for the detection of occult lymph node metastasis in patients with early stage oral cancer. Observational trials (with only neck dissection after positive SLNB) have demonstrated that SLNB is a sensitive method in the detection of occult cervical lymph node metastases. A recent meta-analysis found a pooled sensitivity of 91 % (95 % CI 84–95 %) and a negative predictive value ranging from 92 to 98 % when follow-up was used as reference standard [1]. Long-term follow-up studies showed that SLNB is a safe procedure [2, 3]. Recently, we reported a sensitivity of 93 % and a negative predictive value of 97 % of SLNB in our first 90 early oral cancer patients [4].

Metastatic tumor deposits can be categorized as isolated tumor cells (ITC), micrometastasis, and macrometastasis. ITCs are generally defined as tumor deposits ≤0.2 mm (pN0i+), micro- (pN1mi), and macrometastases (pN1) as tumor deposits of 0.21–2.0 mm and >2.0 mm, respectively. In addition, for ITC, more specific histopathological characteristics have been described: no contact with vessel or lymph sinus wall, no extravasation, no extravascular stromal reaction, and no extravascular tumor cell proliferation [5].

So far, the same strategy has been used in the case of sentinel nodes with ITCs, micro-, and macrometastases, which means a (selective) neck dissection. Broglie et al. found significantly higher hazard ratios in overall disease specific and disease free survival for micrometastases and macrometastases, whereas ITCs were significant determinants for disease specific survival compared with SLN negative patients [6].

A report of a European multicenter study on 109 oral squamous cell carcinoma patients with positive SLNB showed additional (non-SLN) metastases in 34.4 % of the neck dissection specimens [7].

The recent update of this trial demonstrated a statistically lower overall survival for micro- and macrometastases compared with ITC [8]. If a reliable nomogram to predict non-SLN metastases based on degree of metastatic tumor deposits in SLNs can be developed, SLNB might be a therapeutic rather than just a diagnostic procedure, i.e., avoiding subsequent tumor-negative neck dissections. The aim of the present retrospective study and the literature review is to analyze risk factors for the presence of non-SLN metastases in SLNB positive early oral cancer patients.

## Materials and methods

### Retrospective study

From February 2007 until October 2014, 139 consecutive patients with cT1-2N0 squamous cell carcinomas of the oral cavity or oropharynx underwent transoral excision and SLNB. After approval of the Institutional Review Board and Ethics Committee, informed consent was obtained until SLNB was performed as standard procedure in our institution. SLNB was performed according to the EANM/SENT joint practice guidelines [9]. A detailed description of the procedure in our institution had been described previously [4].

A positive SLNB was followed by (selective) neck dissection in 36/37 (97 %) patients (one patient with ITC was treated by radiotherapy only, which was indicated for adverse histopathological findings of the primary tumor).

The neck dissection specimen was histopathologically examined for additional lymph node metastases using a routine procedure (no step-serial sectioning and immunohistochemistry). The presence and localization (level) of additional lymph node metastasis were scored for each patient.

The numbers of tumor positive (1 vs. >1) and negative (0 vs. ≥1) SLNs and their ratio (≤50 vs. >50 %) were scored for each patient.

### Literature analysis

Studies included in recent meta-analyses [1, 10] were analyzed for data on the degree of metastatic involvement of SLN, the status of other SLNs, and additional non-SLN metastases in neck dissection specimens following positive SLNB. In addition, references were explored to identify other relevant articles. If presented (or could be subtracted from the data provided), the rate of positive non-SLN was scored for ITC, micrometastasis, macrometastasis, number of positive SLNs (1 vs. >1), number of negative SLNs (0 vs. ≥1), and their ratio ratio (≤50 vs. >50 %) per patient.

Due to low numbers, no statistical analyses were performed.

## Results

### Retrospective study

At least one histopathologically positive SLN was found in 36/139 (26 %) of patients, yielding a total of 43 positive SLNs. One patient with a paramedian T1 tongue tumor was diagnosed with bilateral positive SLNs. In both neck sides, the largest tumor deposit in the positive SLN, respectively, ITC, and macrometastasis, was separately investigated. The remaining patients with at least two positive SLNs had only unilateral metastasis, and the largest tumor deposit was taken for evaluation and follow-up of the neck. Overall, we analyzed 36 patients with 37 SLN positive neck sides, subdivided into 7 necks with ITC, 14 with micro-, and 16 with macrometastasis (Tables 1, 2).

**Table 1:** Data of demographic and tumor-related patient characteristics

Characteristic	Overall (%)	Status of SLNB	
		Negative (%)	Positive (%)
Patients, *n* (%)	139 (100 %)	103 (74 %)	36 (26 %)
Gender, *n* (%)			
Male	71 (51 %)	54 (52 %)	17 (47 %)
Female	68 (49 %)	49 (48 %)	19 (53 %)
Median age (year) (range)	60 (27–86)	60 (27–85)	62 (29–86)
Tumor location, *n* (%)			
Tongue	86 (61 %)	62 (60 %)	24 (66 %)
Floor of mouth	40 (29 %)	31 (30 %)	9 (25 %)
Buccal mucosa	6 (4 %)	6 (6 %)	0
Inferior alveolar process	4 (3 %)	2 (2 %)	2 (6 %)
Soft palate	3 (2 %)	2 (2 %)	1 (3 %)
Clinical T stage, *n* (%)			
T1	97 (70 %)	81 (79 %)	16 (44 %)
T2	42 (30 %)	22 (21 %)	20 (56 %)
No of SLNs	328	285 (87 %)	43 (13 %)
Follow-up, (*m*) (range)			
Observation time (months)	36 (1–102)	36 (1–102)	36 (1–98)

*SLNB* sentinel lymph node biopsy, *SLNs* sentinel lymph nodes

**Table 2:** Prevalence of ITC, micrometastasis, and macrometastasis in positive SLNs

Study	All	ITC	Micro	Macro
Barzan [11]	2^a^	0 (0 %)	1 (50 %)	1 (50 %)
Mozillo [12]	4	0 (0 %)	4 (100 %)	0 (0 %)
Stoeckli [13]	9	1 (11 %)	5 (56 %)	3 (33 %)
Keski-Säntti [14]	2	0 (0 %)	1 (50 %)	1 (50 %)
Bilde [15]	11	3 (27 %)	6 (55 %)	2 (18 %)
Atula [16]	34	5 (15 %)	14 (41 %)	15 (44 %)
Kovacs [17]	9	0 (0 %)	3 (33 %)	6 (67 %)
Alkureishi [18]	42^b^	0 (0 %)	10 (24 %)	32 (76 %)
Burcia [19]	38	14 (37 %)	15 (39 %)	9 (24 %)
Terada [20]	5	0 (0 %)	3 (60 %)	2 (40 %)
Broglie [6]	42	10 (24 %)	19 (45 %)	13 (31 %)
Present study	36	6 (16 %)	14 (39 %)	16 (44 %)
Total	234	39 (17 %)	95 (41 %)	100 (43 %)

*ITC* isolated tumor cells; *micro* micrometastasis; *macro* macrometastasis; *SLN* sentinel lymph node

^a^Only results of cNO early oral cancer

^b^Definition of micrometastasis: only detected by step-serial sectioning and/or immunohistochemistry

In none of the SLNs with ITC based on size, extravasation, extravascular stromal reaction or extravascular tumor cell proliferation were found, but all these SLNs had contact with lymph sinus wall.

In 6/36 (17 %) patients who underwent a subsequent neck dissection, additional lymph node metastases were found. All patients had T2 tumors and the SLN had contained a macrometastasis (Table 3).

**Table 3:** Prevalence of non-SLN metastases in relation to size of the SLN metastasis, number of positive SLNs, and ratio positive/negative SLNs

Study	All	ITC	Micro	Macro	1 pos SLN	>1 pos SLN	0 neg SLN	≥1 neg SLN	≤50 % SLN pos	>50 % SLN pos
Zitsch [23]	50 % (2/4)		50 % (2/4)		67 % (2/3)	0 % (0/1)	50 % (1/2)	50 % (1/2)	50 % (1/2)	50 % (1/2)
Taylor [24]	0 % (0/4)				0 % (0/3)	0 % (0/1)	0 % (0/1)	0 % (0/3)	0 % (0/2)	0 % (0/2)
Barzan [11]^d^	0 % (0/2)		0 % (0/1)	0 % (0/1)	0 % (0/1)	0 % (0/1)	0 % (0/1)	0 % (0/1)	0 % (0/1)	0 % (0/1)
Civantos [26]	30 % (3/10*)*	0 % (0/2)	38 % (3/8)							
Hoft [22]	25 % (3/12)				33 % (3/9)	0 % (0/3)	50 % (2/4)	13 % (1/8)	13 % (1/8)	50 % (2/4)
Jeong [30]	33 % (2/6)				20 % (1/5)	100 % (1/1)	50 % (1/2)	25 % (1/4)	25 % (1/4)	50 % (1/2)
Stoeckli [13]^b^	56 % (5/9)	0 % (0/1)	40 % (2/5)	100 % (3/3)	50 % (3/6)	67 % (2/3)	67 % (4/6)	33 % (1/3)	33 % (1/3)	67 % (4/6)
Stoeckli [13]^c^	21 % (4/20*)*	0 % (0/5)	11 % (1/9)	50 % (3/6)	14 % (2/14)	33 % (2/6)	20 % (1/5)	20 % (3/15)	15 % (2/13)	29 % (2/7)
Bilde [15]	9 % (1/11)	33 % (1/3)	0 % (0/6)	0 % (0/2)	11 % (1/9)	0 % (0/2)	0 % (0/1)	10 % (1/10)	11 % (1/9)	0 % (0/2)
Tartaglione [33]	25 % (2/8)				25 % (2/8)	0 % (0/0)	100 % (1/1)	14 % (1/7)	14 % (1/7)	100 % (1/1)
Atula [16]	39 % (13/33)	20 % (1/5)	46 % (6/13)	40 % (6/15)	43 % (6/14)	20 % (1/5)^a^	50 % (3/6)	31 % (4/13)^a^	33 % (4/12)	43 % (3/7)
Kovacs [17]	11 % (1/9)		0 % (0/3)	17 % (1/6)						
Broglie [6]	14 % (6/42)	20 % (2/10)	13 % (4/32)							
Rigual [37]	75 % (3/4)				50 % (2/4)	0 % (0/0)	100 % (2/2)	50 % (1/2)	50 % (1/2)	100 % (2/2)
Present study	17 % (6/36)	0 % (0/6)	0 % (0/14)	38 % (6/16)	13 % (4/31)	40 % (2/5)	15 % (2/13)	17 % (4/23)	14 % (3/22)	21 % (3/14)
Total	24 % (51/210)^e^	13 % (4/32)	20 % (11/55)	40 % (19/49)	24 % (26/107)	29 % (8/28)	40 % (17/44)	19 % (18/91)	19 % (16/85)	38 % (19/50)
			26 % (37/144)							

*SLN* sentinel lymph node; *ITC* isolated tumor cells; *micro* micrometastasis; *macro* macrometastases; *pos* positive; *neg* negative

^a^Only for five patients specific data available

^b^Only feasibility and validation phase

^c^Clinical application phase update by Broglie et al. (2012)

^d^Only cNO early oral cancer

^e^Rate based on studies presented in Table 3. In general, the prevalence of non-SLN metastases in all 26 studies analyzed including our study was 31 % (156/511)

Additional non-SLN metastases were found in level I (*n* = 3), level III (*n* = 6), level IV (*n* = 1), and level V (*n* = 1). In one patient, non-SLN metastasis was restricted to the same level as the positive SLN, in one patient in adjacent and non-adjacent levels, and in 4 patients, non-SLN metastasis were only found in non-adjacent levels.

If >1 SLN was positive, 2/5 (40 %) of the patients had additional neck metastases compared to 4/31 (13 %) in patients with a single positive SLN. In 2/13 (15 %) patients with solely positive SLN(s), additional non-SLN metastases were found (vs. 17 % if synchronous presence of negative SLNs was present). If more positive than negative SLNs were present (>50 % SLN positive), additional non-SLN metastases were found in 3/14 (21 %) patients compared to 3/22 (14 %) if a similar or higher number of negative than positive SLNs were found (Table 3).

### Review of the literature

Eleven studies [6, 11–20] had categorized the size of tumor deposits in SLNs. Including the data from our study, ITC was present in 17 % of 234 patients (range 0–37 %), micrometastasis in 41 % (19–100 %), and macrometastasis in 43 % (0–76 %) (Table 3). Additional non-SLN metastases were mainly found in levels I, II, and III and sometimes in level IV or V [7, 13, 15, 16, 21, 22]. The pooled prevalence of non-SLN metastasis in patients with positive SLN(s) of this study and 26 other studies [6, 7, 11–17, 21–37] was 31 % (156/511).

The pooled probability of non-SLN metastasis in this present study and 8 other studies [6, 11, 13, 15–17, 23, 25] was 13 % (4/32), 20 % (11/55), and 40 % (19/49) for ITC, micro-, and macrometastases, respectively. This probability was 26 % (37/144) for micro- and macrometastases combined.

Including our results, a higher pooled prevalence for additional non-SLN metastases had been found when >1 positive SLNs were present (29 vs. 24 %) [11, 13, 16, 22–24, 30], the absence of negative SLNs (40 vs. 19 %) [13, 15, 16, 22–24, 30, 33, 37], and in the case of a positive SLN ratio of more than 50 % (38 vs. 19 %) [13, 16, 22–24, 30, 33]. Results are shown in Table 3.

## Discussion

Patients with positive SLNB undergo generally subsequent (completion) neck dissection, because there is no reliable means of detecting or predicting non-SLN metastasis. SLNB is associated with significant less morbidity than elective neck dissection and identification of patients who do not benefit from subsequent neck dissection may decrease this morbidity even further [38].

The prediction of the presence of non-SLN metastasis in the neck after positive SLNB can theoretically be improved in two ways: dividing the tumor deposits in SLNs into subgroups or adding other predictive factors in a risk profile.

Combining this study with our analysis of the literature, we found an inverse relation between the size of tumor deposits in the SLN and the probability of a non-SLN: 13 % for ITC, 20 % for micrometastasis, and 40 % for macrometastasis. Since the prevalence of non-SLN metastasis in the neck dissection specimen following ITC in SLNs is substantial, in early stage oral cancer, one cannot refrain from neck dissection after any category of positive SLNB. When patients with a low risk of non-SLN metastasis can be identified, a wait and scan policy using USgFNAC may be justified [39].

The commonly used definition of isolated tumor cells is based on size (0.2 mm or less) rather than designation of the metastatic tumor deposit. ITC is then considered to be a small micrometastasis “waiting to grow” (precursor of micrometastasis) with a risk these necks with SLNs containing ITC may also harbor micro- or macrometastases [40]. In this study, all ITCs based on size had the same morphologic features: no extravasation, extravascular stromal reaction or extravascular tumor cell proliferation, but all had contact with lymph sinus wall. Since these deposits seem to be real ITC, this latter feature is debatable.

Review of the literature revealed that only a limited number of small studies classified SLN tumor deposits in ITC, micrometastasis, and macrometastasis. The wide variety of rates of the different categories in our literature review may reflect the lack of uniformly used definitions.

Consequently, these numbers are too low to perform reliable statistical analyses on the risk of non-SLN metastases in these different tumor deposits in SLNs. To explore if patients with ITC or micrometastasis in SLNs need a subsequent neck dissection, it is important that all future studies report SLN metastases in these categories. Only, then, the question if SLNB can be used as treatment, and not only as diagnostic procedure, in patients selected by the type of tumor deposit in SLNs can be answered.

In breast cancer, SLNB is accepted as standard diagnostic technique for clinically node negative patients. Complete axillary lymph node dissection is generally recommended if the SLNB is positive. Non-SLN metastases are detected in 35–50 % of SLN positive patients. Only some series report that the prevalence of ITC and distinction between ITC and micrometastasis could be difficult [41, 42]. The reported rate of micrometastasis as the largest tumor deposit in SLN positive breast cancer patients varies considerably: from 24 to 93 % [43]. In patients with tumor deposits in SLNs, the prevalences of ITC, micrometastasis, and macrometastasis are 7–16, 16–32, and 58–78 %, respectively. Non-SLN metastases are found in 0–13, 12–27, and 48–50 % in patients with ITC, micro-, and macrometastases in SLNs, respectively [44–49].

Different nomograms in predicting non-SLN metastases in breast cancer patients with a positive SLNB have been developed, usually including the largest detected size of SLN metastasis and the proportion of involved SLNs among all removed SLNs [43]. The treatment strategy for micrometastasis in SLN is under debate. It has been suggested to refrain patients with ITC in their SLN from axillary lymph node dissection [44–46]. A recent review including 7151 breast cancer patients with positive SLNB in whom an axillary lymph node dissection was omitted revealed an axillary recurrence rate of 0.7 % (range 0–7.1 %) for macrometastasis and 0.3 % (range 0–3.4 %) for micrometastasis and ITC. Unfortunately, micrometastasis and ITC could not be analyzed separately, and details regarding adjuvant treatment were lacking in the majority of studies [50]. Since breast cancer patients are often treated with adjuvant systemic therapy, these strategies cannot easily be translated to early oral cancer patients who are usually treated with surgery as monotherapy.

A meta-analysis of predictive factors for non-SLN metastases in breast cancer patients with a positive SLN confirmed a high likelihood of non-SLN metastases for size of SLN metastasis of more than 2 mm [macrometastasis; odds ratio (OR) 4.22], extracapsular extension in the SLN (OR 4.10), one or less negative SLN (OR 2.66), more than one positive SLN (OR 2.60), tumor size >2 cm (OR 2.41), a ratio of positive SLN of more than 50 % (OR 2.25), and lymphovascular invasion (OR 2.24) [51]. Recently, the same authors developed a risk score based on these parameters [52].

In oral oncology, Gurney et al. [7] reported other predictive factors for the presence of non-SLN metastases in SLNB positive necks: tumor site (higher risk as the primary tumor was located at the posterior part of the oral cavity), increased stage (T2–4 stage at higher risk), and number of negative SLNs (lower risk in higher number of negative SLNs). In this study, all patients with non-SLN metastases had T2 oral squamous cell carcinoma. Although tumor thickness or depth of invasion [53] and molecular markers [54] has predictive value for the presence of (occult) lymph node metastasis, their role in predicting the presence of non-SLN metastasis in oral cancer patients with a positive SLNB is not known yet.

Our retrospective study suggests if both a positive SLN and a negative SLN are present the prevalence of non-SLN metastases seems nearly equal compared to patients with solely positive SLNs, in contrast to other studies (Table 3). Since distinguishing real SLNs from the second echelon nodes may be difficult [55], it can be anticipated that (some of) these negative SLNs may be, in fact, the second echelon nodes. If more positive than negative SLNs are present, the probability of non-SLN metastases seems to be higher, also in case of a ratio of positive SLNs of more than 50 %. Due to the low number of cases, statistical analysis could not be performed and more larger studies are needed to confirm these ideas.

A large multicenter study showed in 1/122 neck dissections following positive SLNBs of early oral cancer non-SLN metastases in levels other than I–III [7]. These non-SLN metastases had been found in 15 % of the patients in the same level, in 17 % in an adjacent level, and in 2 % in a non-adjacent level. In our retrospective study, all non-SLN metastases were found in levels I–IV except one in level V. In this latter patient, two positive SLNs and five additional non-SLNs were found. In 67 % (4/6) of the patients, non-SLNs were only found in non-adjacent levels. If the future studies report on the level involved by non-SLN metastases, more tailored (super)selective neck dissections may be defined.

Analysis of the literature, including our present study, showed that additional non-SLN metastases were found in 31 % of neck dissections following positive SLNB. Selected by tumor deposit, these percentages were 13 % for ITC, 20 % for micro-, and 40 % for macrometastasis in SLNs. This prevalence may be underestimated, since, in most studies, non-SLNs are examined using routine histopathological examination without step-serial sectioning and immunohistochemistry. Studies on neck dissection specimens show that immunohistochemistry can reveal small metastases in 15 % of the patients that remain undetected in routine H&E staining [56].

Reporting other risk factors may be useful to develop a nomogram selecting SLNB positive patients for neck dissection and active surveillance or wait and scan follow-up. The presence of more than one positive SLN, the absence of negative SLNs (besides a positive SLN), and a positive SLN ratio of more than 50 % may be predictive factors for non-SLN metastasis in SLNB positive patients. To this point, there is no well-argued reason to refrain from an additional neck dissection based on these risk factors or tumor size in the SLN. The presented data support the use of a selective neck dissection, when because of an SLNB positive neck an additional neck dissection is indicated.
